# Supplementary material for: Signal Peptide Efficiency: From High-Throughput Data to Prediction and Explanation
Source: ACS Synth Biol. 2023 Jan 17;12(2):390–404. doi: 10.1021/acssynbio.2c00328 (PMC9942255; doi:10.1021/acssynbio.2c00328)
Supplement: Supplementary file 1 — sb2c00328_si_001.pdf [file sb2c00328_si_001.pdf]

# Supporting Information

## Signal Peptide Efficiency:
## from High-throughput Data to Prediction and Explanation

*Stefano Grasso [1,2,†], Valentina Dabene [3,4,†], Margriet M.W.B. Hendriks[2], Priscilla Zwartjens[2], René Pellaux[4], Martin Held[3], Sven Panke[3], Jan Maarten van Dijl[1,#,*], Andreas Meyer[4,#], Tjeerd van Rij[2,#,*]*

*[†,#] These authors contributed equally*

[1] University of Groningen, University Medical Center Groningen, Department of Medical Microbiology, Hanzeplein 1, 9700 RB Groningen, the Netherlands

[2] DSM Biotechnology Center, Alexander Fleminglaan 1, 2613 AX Delft, Netherlands

[3] ETH Zurich, Department of Biosystems Science and Engineering, Mattenstrasse 26, 4058 Basel, Switzerland

[4] FGen AG, Hochbergerstrasse 60C, 4057 Basel, Switzerland

* To whom correspondence should be addressed:

TvR: Tel: +31628441843; Email: tjeerd.rij-van@dsm.com

JMvD: Tel: +31503615187; Email: j.m.van.dijl01@umcg.nl

SG's Present Address: Lesaffre International, 101 Rue de Menin, 59700 Marcq-en-Barœul, France

**Running title**: Prediction of signal peptide efficiency in *Bacillus*

**Supplementary Figure S1. Optimization of NLR culture conditions. (a)** Bright-field (top) and fluorescence microscopy (bottom) images of NLRs with embedded *B. subtilis* DB104 strains producing AmyQ without any SP (i.e. negative control, NC) or producing and secreting AmyQ with its native SP (i.e. positive control, PC). The NC was cultured in NLRs in growth medium supplemented with amylopectin (0.5% (w/v)) for 16 h, while the PC was cultured either under the same conditions as the NC (with amylopectin, PC +), or in the growth medium only (i.e. PC -). After cultivation of the NC in the NLRs, all hydrogel beads, empty and occupied (red circles in upper panel) showed a similar green fluorescence intensity, presumably because the strain did not secrete any amylase and therefore there was no fluorescein-starch degradation independent of NLR occupation. In the PC- case, the fluorescein-starch was instead completely degraded in both the occupied (red circles) and empty NLRs, presumably due to the high secretion of AmyQ, its rapid diffusion among NLRs, and fluorescein-starch degradation throughout the entire population. In the PC + case, by adding amylopectin to the medium, a clear distinction between the NLRs embedding the PC (red circles in upper panel) and the empty NLRs can be seen: the NLRs harboring a colony lost the fluorescein-starch due to AmyQ secretion, while diffusion between NLRs was reduced due to high amylopectin levels in the medium. This implies that any enzyme released from the beads will first bind and hydrolyze the amylopectin in the medium, minimizing degradation of starch in proximal NLRs. The exposure time applied for fluorescence microscopy was 500 ms for the NC and the PC + samples, while a 700 ms exposure was applied for the PC -. Scale bar: 200 µm. **(b)** Green fluorescence profile of the NLRs from the NC and the PC + samples. As already visible in the microscopy images in (a), the PC + showed two populations, representing empty and occupied NLRs, with a 5-fold difference in the mean value. In contrast, in the NC histogram it is not possible to distinguish NLRs with NC microcolonies from the empty NLRs. The comparison of the two histograms highlights the importance of selecting an appropriate time window for the analysis. The baseline (i.e. empty NLRs) in the PC + sample is shifted towards lower fluorescence values, as some AmyQ escapes the diffusional limitation (note that in this experiment all occupied reactors contain high-secreting colonies) and leads to a background degradation of fluorescein-starch in non-occupied NLRs.

**Supplementary Figure S2. Comparison of the NLR- and MTP-based amylase assays for a random selection of 95 clones from the SP-library.** The abscissa marks results from the MTP assay, with a value of 1 for the efficiency of the native SP of AmyQ; the ordinate marks weighted average (WA) values from the NLR assay. Of note, data points with a WA between 5 and 10 could not be measured with the standard MTP assay due to its low sensitivity; an optimized MTP assay and a hydrolysis test on starch agar plates was performed for the poorly secreting variants (Supplementary Figure S4 and Supplementary Table S3). Error bars represent the standard error of the mean over two replicates for the MTP-based assay; error bars are not represented for the NLR-based assay because their size was comparable to that of the marker. The dashed red regression trend line is based on all 95 data points (i.e. including those that could not be measured in the MTP-assay).

**Supplementary Figure S3. Hydrolysis test on starch agar plates of the poorly secreting variants from Supplementary Figure S1 and Supplementary Table S3.** Strains, which showed activity in the NLR-based assay but for which no secretion activity could be determined using the MTP assay (data on the ordinate in Supplementary Figure S1 and in Supplementary Table S3), were further characterized using the starch hydrolysis test on agar plates. The images show clear degradation zones around the colonies for the majority of the variants tested, suggesting that these clones showed secretion activities too low to be measured in the MTP assay, but detectable by the NLR-based assay. This analysis demonstrates that the NLRs-based enzymatic assay has a much higher dynamic range and improved sensitivity compared to the MTP analysis.

**Supplementary Figure S4. Principal component analysis (PCA)**. PCA was performed over three datasets: in green the 134 wild-type known SPs from *Bacillus subtilis*; in red the whole dataset describing the designed library of 11,643 unique SPs; in blue the dataset with the 4,421 reliably measured SPs (Train + Test sets). On the ordinate the cumulative variance explained by the corresponding number of components, reported on the abscissa, is shown. For all of the three datasets a total of 156 features, the same employed in the ML model, was taken into account. It is possible to notice how, for the whole dataset and for the Train + Test set, a higher number of components is needed to explain the same amount of variance, if compared to the wild-type SPs. To explain the entire variance 156 components are needed for the whole dataset and for the Train + Test set, while only 135 components are necessary to explain the whole variance in the wild-type SPs dataset.

**Supplementary Figure S5. Explanation of the applied SHAP methodology**. **(a)** SHAP is a method to interpret machine learning models based on Shapley values and on the game theory. Given a machine learning model, which can be considered as a black box due to its intrinsic lack of interpretability, SHAP can provide an explanation for each sample that can be fitted by the model, regardless whether it was used to train and test the model or whether it was a novel sample. **(b)** Illustration of the basic SHAP mechanism. Using a reference dataset (i.e. the unified train and test sets) and the built machine learning model, SHAP calculates a SHAP values matrix of the same size and shape of the reference dataset. When inspected sample by sample, SHAP values provide local explanations, allowing to understand the prediction over a specific sample; when instead SHAP values from the whole dataset are combined, they provide a global explanation, allowing to fully explain the machine learning model.

**Supplementary Figure S6. Second order interactions between features. (a)** SHAP dependence plot for 'GRAVY_SP' (same as Figure 3c). Note that a certain amount of vertical dispersion is due to the interactions between 'GRAVY_SP' and other features. In particular, the negative effect of low hydrophobicity values on secretion efficiency can be enhanced or reduced by other features. These second order interactions are captured by SHAP interactions. **(b)** SHAP dependence plot of the main effect of 'GRAVY_SP'. This plot is similar to the previous one, but it lacks the vertical dispersion caused by the other features. It shows the effect of 'GRAVY_SP', called main effect and plotted on the ordinate, as if it were independent from the other features. **(c)** SHAP dependence plot of the interaction between 'GRAVY_SP' and '-1_A'. The feature interacting most significantly with 'GRAVY_SP' is '-1_A' (see Supplementary Figure S8). Interaction values, plotted on the ordinate, are to be summed to the main effect values from (b) in order to obtain the SHAP value as shown in (a). The plot thus shows how, despite a good degree of hydrophobicity ('GRAVY_SP' around 1.0), not having an Ala in position -1 has a slightly negative impact on the main effect of 'GRAVY_SP'; in fact, it adds approximately 0.2 to the positive effect on secretion efficiency (represented by a main effect value of approximately -0.5, in (b)). On the contrary, the presence of an Ala in position -1 adds a negative value (i.e. a positive impact on secretion efficiency) to the already positive impact of the hydrophobicity. **(d)**, **(e)**, and **(f)** respectively display as well the overall effect of 'Q_Ac', its main effect and its interaction with the feature 'A_C'. The interaction between these two features is the strongest interaction in the model (see Supplementary Figure S8). **(g)**, **(h)**, and **(i)** highlight the same interaction between 'Q_Ac' and 'A_C', but this time shown from the perspective of 'A_C'.

**Supplementary Figure S7. SHAP interaction plot.** In this plot interactions between the 10 most impactful features (see Figure 3b) are represented. On the diagonal of the plots from top-left to bottom right, the main effect of each feature is represented. The main effect is the impact on the model to be attributed to a specific feature, as if it were not interacting with any other. Off-diagonal, the proper interaction effects are represented. Interaction effects capture the influence of one feature on another, and thus cause the vertical dispersion displayed in Figure 3c and Supplementary Figure

S6 (a), (d), and (g). The overall SHAP value, as represented for instance in Figure 3b, is the result of the sum of main effect values for a specific feature and all its interaction values with the other features (see also Supplementary Figure S6).

**Supplementary Figure S8. SHAP summary plot for main effects and interactions.** The plot shows the same type of information as presented in Supplementary Figure S7, but ordered by impact on the model. The plot shows how main effects tend to be more impactful on the model compared to interactions (denoted as {feature}-{feature}, with '*' indicating to which feature the color scale refers to). The most impactful main effect belongs to 'GRAVY_SP', while the most impactful interaction occurs between 'Q_A' and '-1_A' (see also Supplementary Figure S8). Note how some features show relevant interactions, such that their main effect can be less relevant than their overall effect (e.g. '-1_A', or 'CAI_RSCU_SP'), while other features show few interactions, resulting in an impactful main effect (e.g. 'flexibility_N') (compare the order of features with Figure 3b).

**Supplementary Figure S9. Plasmid map of pSG01.** pSG01 was built from pCS75 by removing the insert between the EagI and PmeI sites, and inserting between these two restriction sites the gBlock G1 (Supplementary Table S4), containing the mature part of AmyQ and two BsmBI restriction sites (highlighted) to be used as cloning sites in downstream applications. Due to the specific design, these BsmBI sites will not present in pCS75 derivatives with cloned SPs, while the third BsmBI restriction site is exploited to provide a linear vector for efficient transformation in *B. subtilis*. The sequence encoding the mature part of AmyQ, used as reporter protein, is represented in orange; the transcriptional terminator in red; the three antibiotic resistance genes in violet (Spec, spectinomycin; erm, erythromycin, bla, beta-lactamase); the regions used for genome integration into the *amyE* gene of *B. subtilis* DB104 in light blue; and the cre-lox recombination sites in gray.

**Supplementary Table S1. Signal peptide sequences and feature description. Tab: 'WT sequences.** List of the 134 WT SP sequences used as a starting point for the design of the SP-library with all the respective information provided. **Tab 'Library_w_Bins_and_WA'** lists the sequences used in this study with their descriptive information, including normalized, absolute and relative read counts from NGS data, and the WA score for each sequence. **Tab 'Feature Description':** Explanation of and calculation method for the features used to describe each SP. Here, features are represented only once, but they occur up to 5 times, as features are repeated for each region and for the whole SP; full feature names will thus be 'Feature name'_'region (i.e. N, H, C, Ac, SP)'.

**Supplementary Table S2. Overview of feature editing, processing and clustering. Tab 'Edited Features':** Summary of features and SP regions that were modified in the SP-library to be screened with relative target levels for each region. **Tab 'Feature processing':** For each region (N, H, C, Ac, SP), all the features are displayed. Additionally, those features considered confounding and a priori removed are marked in red, while those removed after clustering are marked in yellow. The remaining features were used to create the Random Forest Regressor model, together with 40 Boolean variables describing the residue present in position -1 and -3 from the cleavage site. **Tab 'Clusters':** Results of the affinity propagation clustering. For each cluster the centroid is highlighted in yellow. Of note: all features of cluster 8 were used in the study, since they were considered as an outgroup; for cluster 10, in addition to the centroid, also the feature 'CAI_RSCU_SP' was included as potentially interesting to be studied on its own.

**Supplementary Table S3. Summarized report of mapped reads and retrieved SP variants across the 10 bins, and the two controls.** The total number of reads refers to the number of valid reads after merging the paired-ends. Note that up to 90% of the SP-library was successfully introduced into *B. subtilis*, but that no more than 63% of the overall SP variants was retrieved during the experiment.

**Supplementary Table S4. SP validation. Tab 'PRE_edit_SPs_info':** List of 60 SPs already assayed and selected to be used in the model validation. **Tab 'POST_edit_SPs_info':** List of the 92 SPs used in the model validation. VC_0 to VC_59 represent modifications of the SP sequences from the 'PRE_edit_SPs_info' tab, while SPs from V_60 to V_91 are *de novo* designed through a pseudo-random approach. **Tab 'Comparison':** Comparison of the 60 modified SPs with their measured and predicted scores, both before and after the modifications.

**Supplementary Table S5. Oligonucleotide sequences and plasmids. Tab 'Primer_list':** List of primers used in this study with the respective sequence and usage. **Tab 'Plasmid_list':** Description of the plasmids used and synthesized in this study. **Tab 'gBlocks list':** all gBlocks used in this study. **Tab 'Bin_Barcodes':** Barcodes used to identify the various bins during the multiplexed sequencing. They are embedded in primers P3-P14.

**Supplementary Table S6. MTP amylase assay of 95 clones randomly picked from the 4,421 analyzed SPs variants.** The results of the assay as described in the Online Methods section 'Assay and ML model validation' are plotted in Supplementary Figure 2. To evaluate the performance of SP variants directing marginal levels of secreted amylase activity (72 out of 95), the incubation time of the assay was extended to 90 min (i.e. sensitive version) for enhanced assay sensitivity. The activity of about 15 additional variants was verified (i.e. Abs>0.1), but activity of 57 clones could not be measured using the MTP assay. As the NLR-based assay showed positive secretion for all picked variants (i.e. WA<9), the amylase activity of these variants (marked by the orange box) was further investigated using the starch hydrolysis test on agar plates (see Supplementary Figure 3).

**File S1. Interactive SHAP force plot for the whole train and test set.** The plot is a horizontal stacking of force plots of single SPs, but rotated of 90 degrees compared to Figure 3c. By selecting ‘model output value’ on the ordinate, it is possible to visualize a summary of all predictions for the whole dataset of 4903 SPs; in addition, hoovering over the plot an explanation of the main features impacting the output is displayed, and values on the axes are highlighted. The model output value is designated by the line separating favorable and detrimental features (i.e. orange and gray areas). Selecting instead on the ordinate one feature effect (e.g. ‘GRAVY_SP’ effects) and on the abscissa values for the same feature (e.g. ‘GRAVY_SP’) it is possible to obtain a plot similar to that in Figure 3b. In this plot it is evident which values of e.g. ‘GRAVY_SP’ are favorable and which are detrimental, according to the presented model. Similarly, by selecting on both axes ‘Length_SP’ it is possible to visualize how 22 AA long SPs are predicted to have a lower secretion efficiency compared to SPs with lengths of 31-35 AAs. The reader is invited to explore the model through this tool.

**File S2. Interactive SHAP force plot for the 59 SPs used to validate the model in their original form** (i.e. as present in the SP-library). By selecting ‘model output value’ and ‘original sample ordering’ the two groups of manually edited SPs are visible. We recommend using this interactive tool only to explore the features of the selected SPs, and their modifications comparing this with File S3, but not to explore the model.

**File S3. Interactive SHAP force plot for the 59 SPs used to validate the model in their engineered form**. By selecting ‘model output value’ and ‘original sample ordering’ the two groups of manually edited SPs are visible (they will be opposite compared to File S2). We recommend using this interactive tool only to explore the features of the selected SPs and their modifications compared to File S2, but not to explore the model.
