# Supplementary figures and images for: Signal Peptide Efficiency: From High-Throughput Data to Prediction and Explanation
Source: ACS Synth Biol. 2023 Jan 17;12(2):390–404. doi: 10.1021/acssynbio.2c00328 (PMC9942255; doi:10.1021/acssynbio.2c00328)

**a**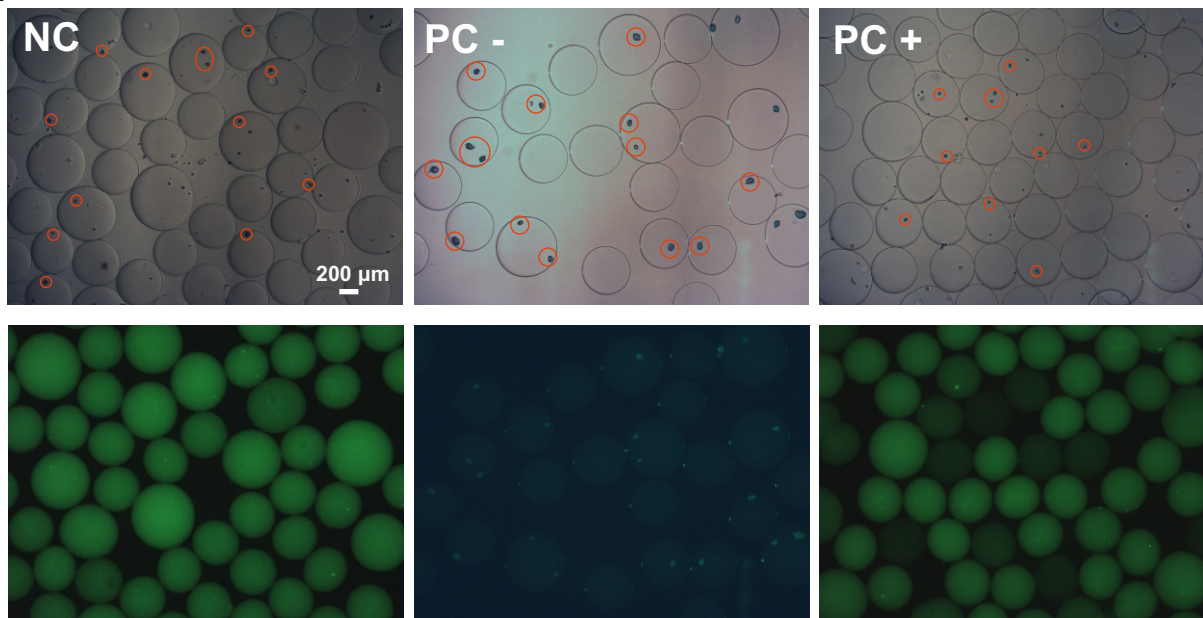**b**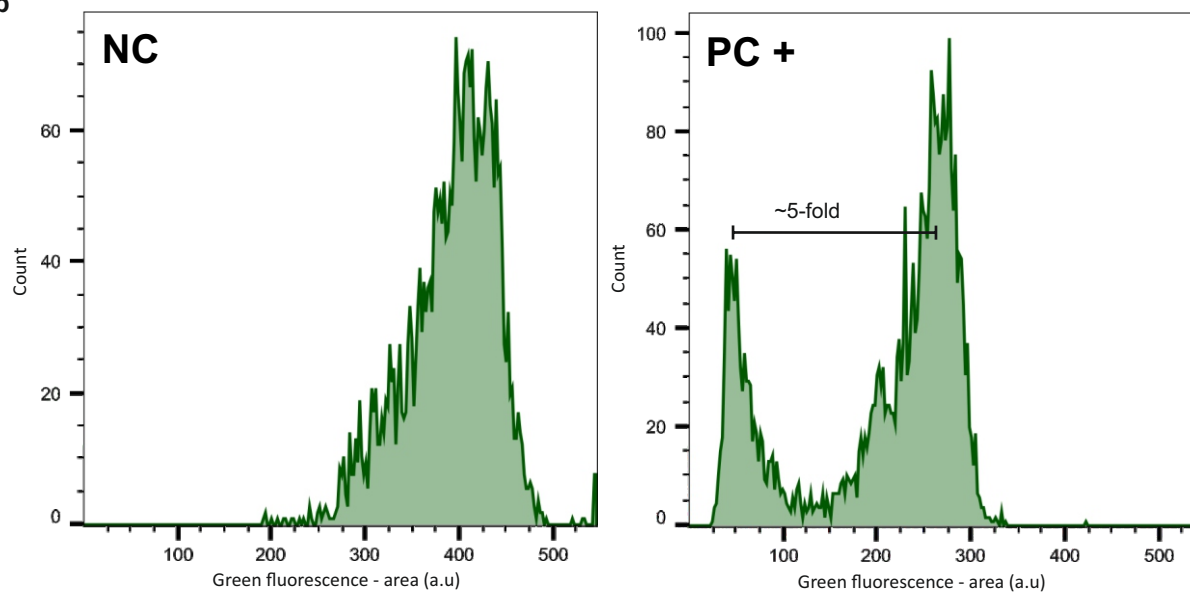

Supplement: Supplementary file 2 — sb2c00328_si_002.pdf [file sb2c00328_si_002.pdf]

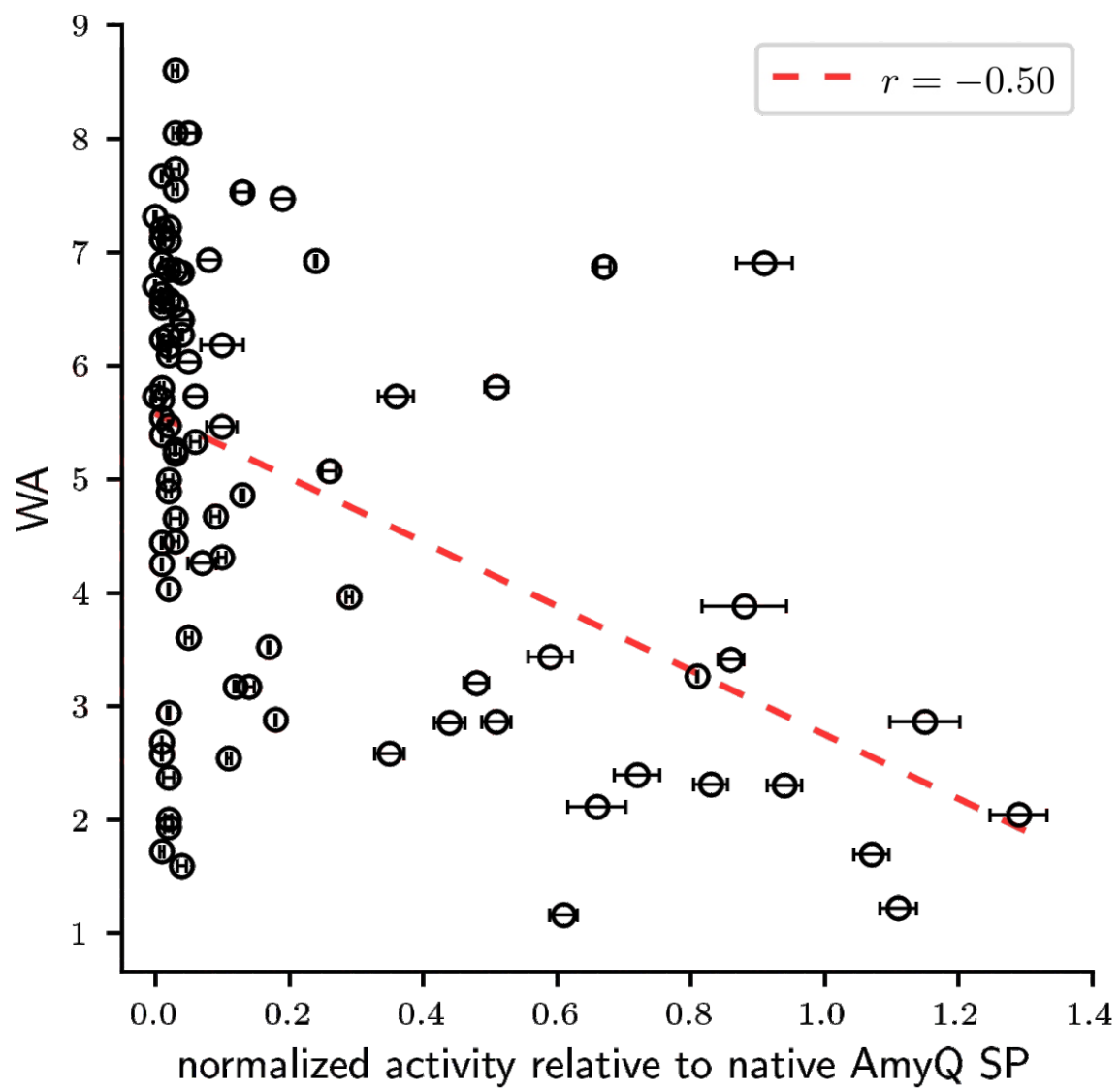

Supplement: Supplementary file 3 — sb2c00328_si_003.pdf [file sb2c00328_si_003.pdf]

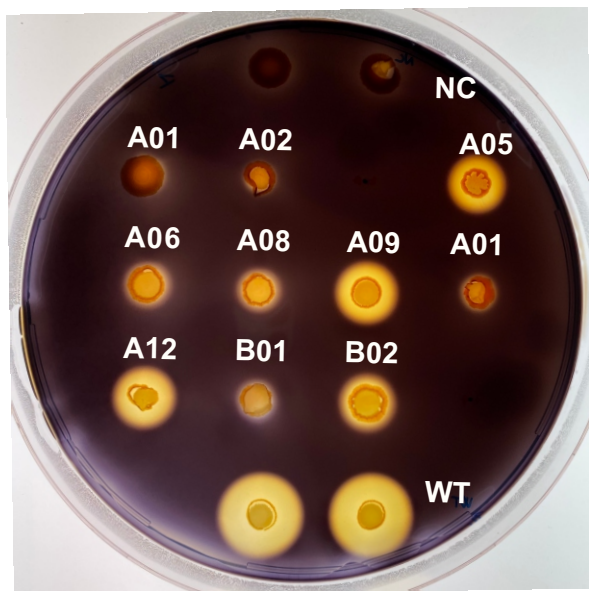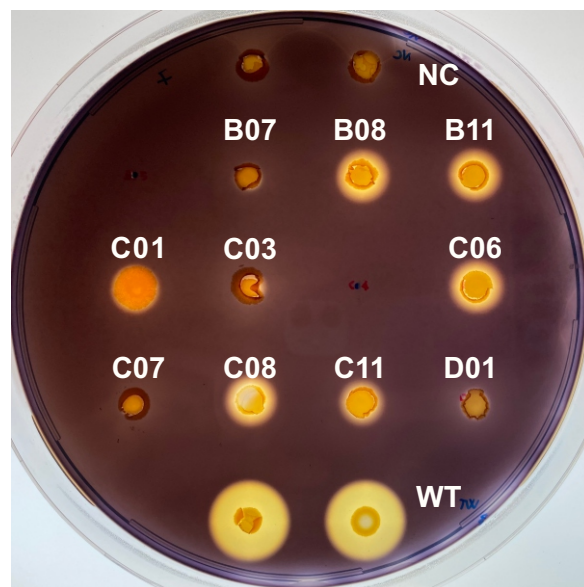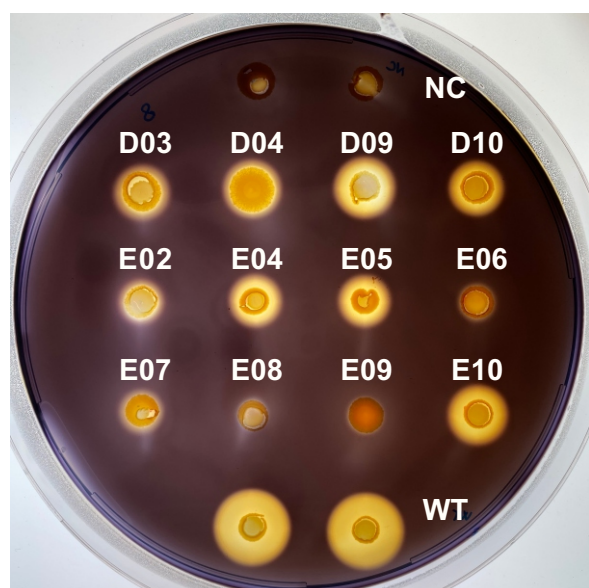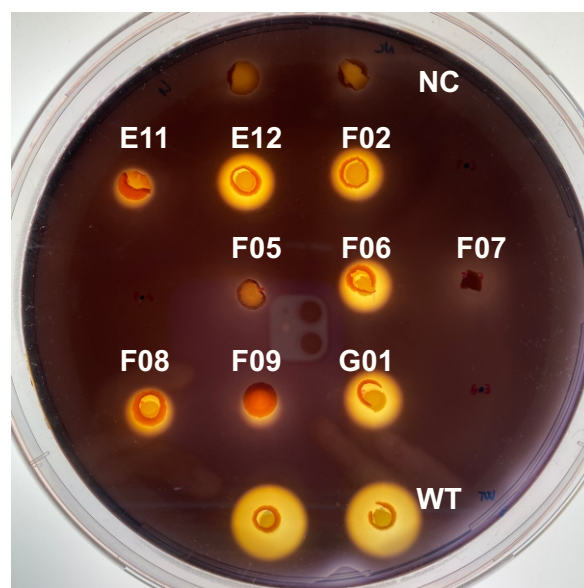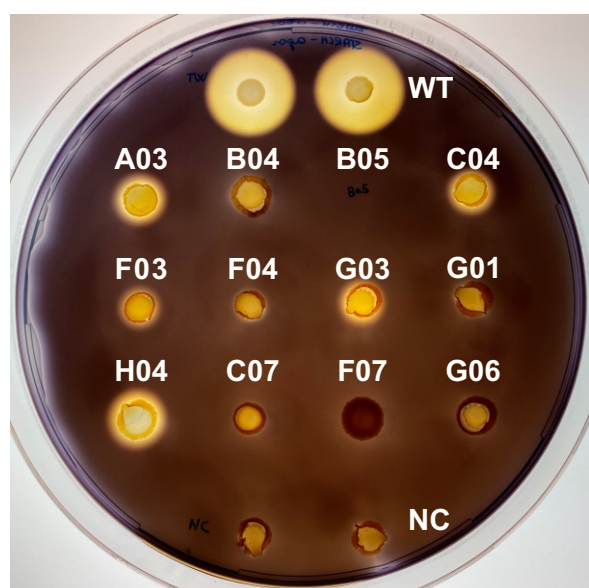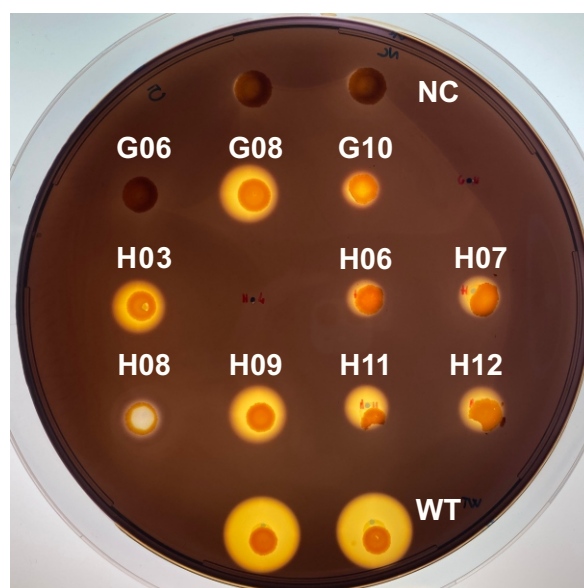

Supplement: Supplementary file 4 — sb2c00328_si_004.pdf [file sb2c00328_si_004.pdf]

Principal component analysis of the different datasets

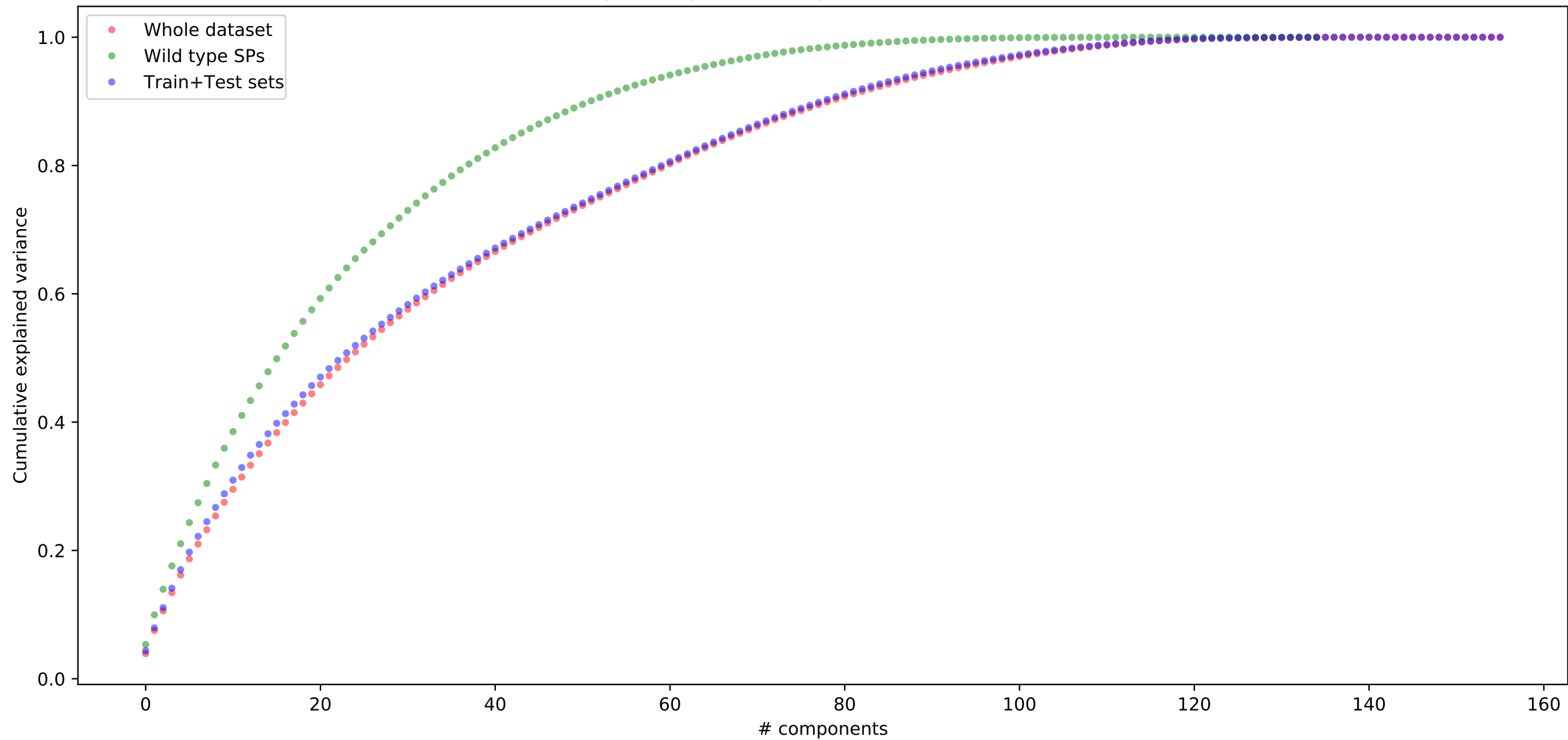

Supplement: Supplementary file 5 — sb2c00328_si_005.pdf [file sb2c00328_si_005.pdf]

a

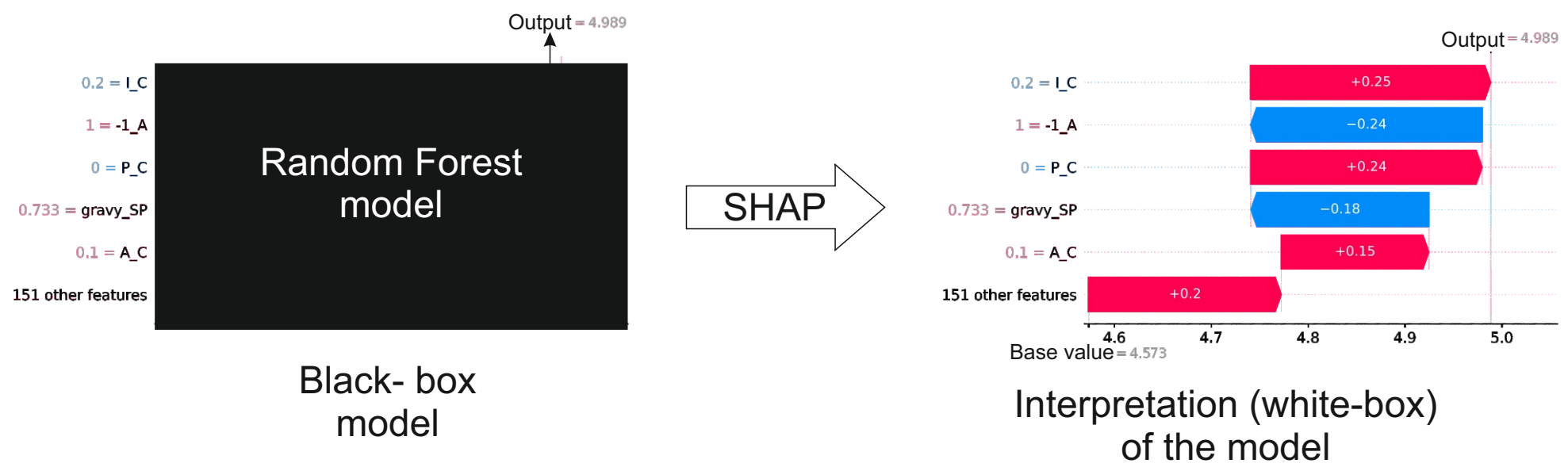

b

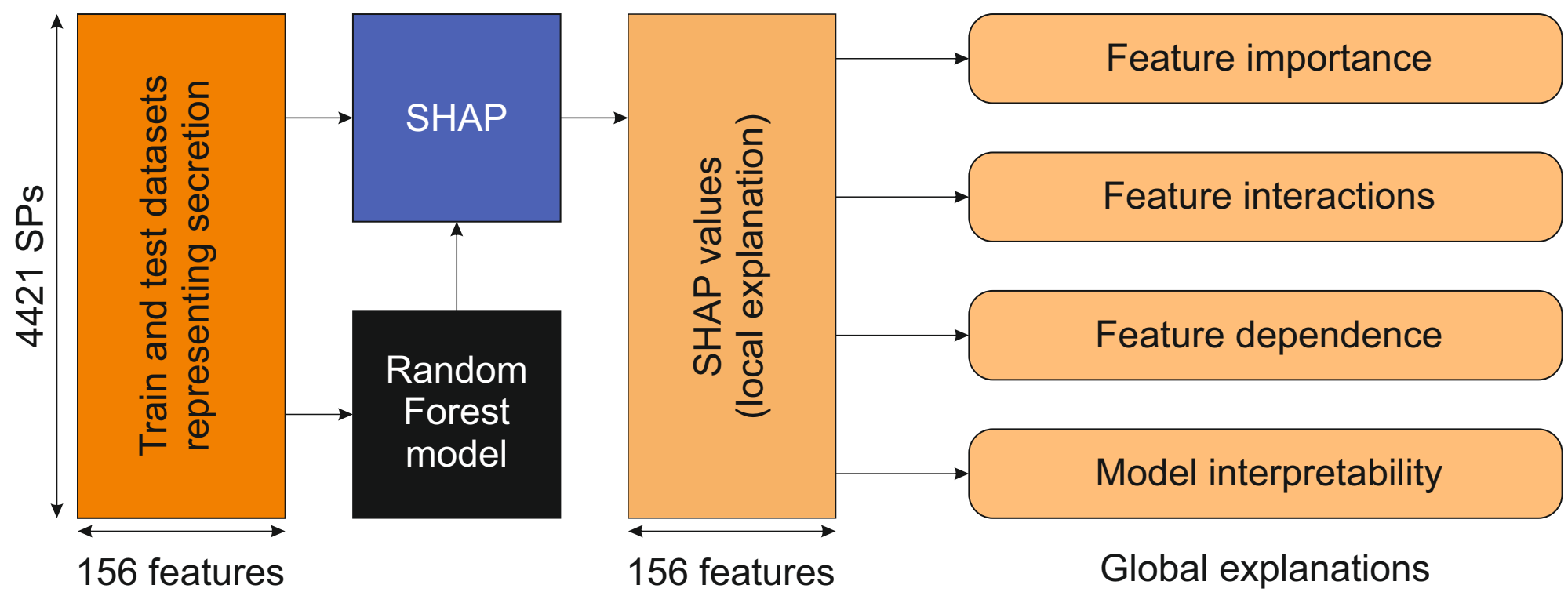

Supplement: Supplementary file 6 — sb2c00328_si_006.pdf [file sb2c00328_si_006.pdf]

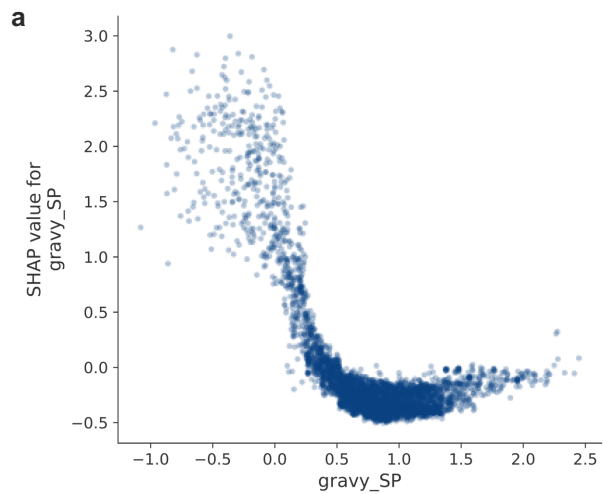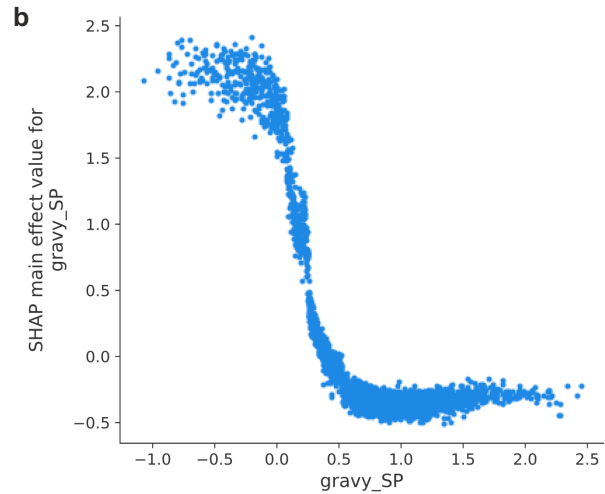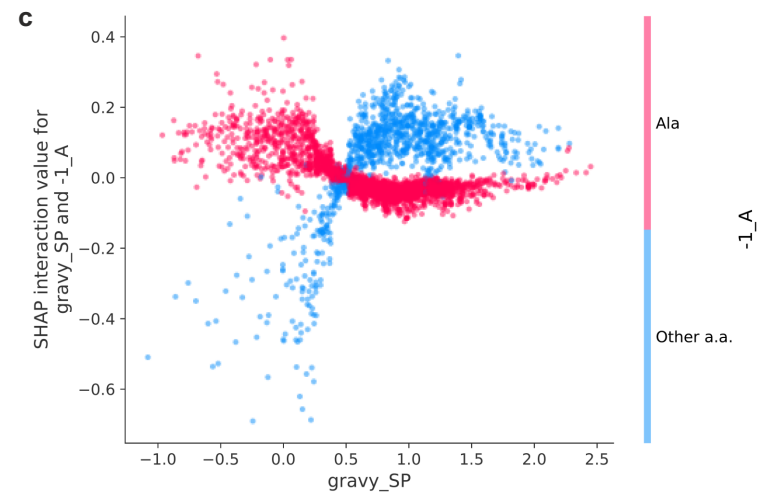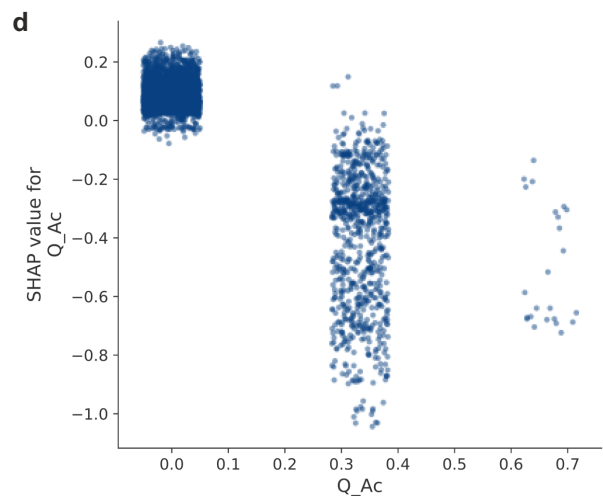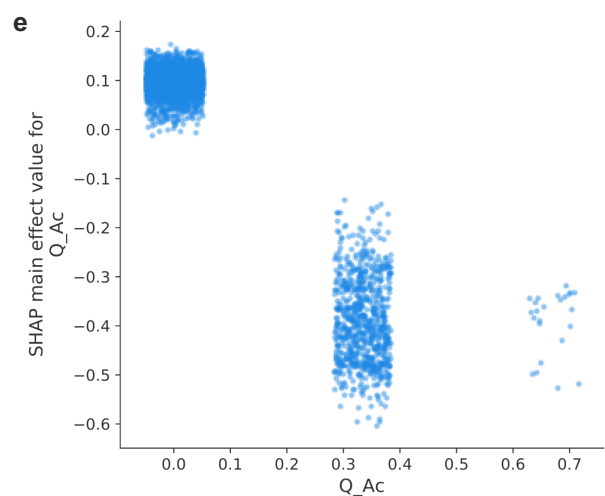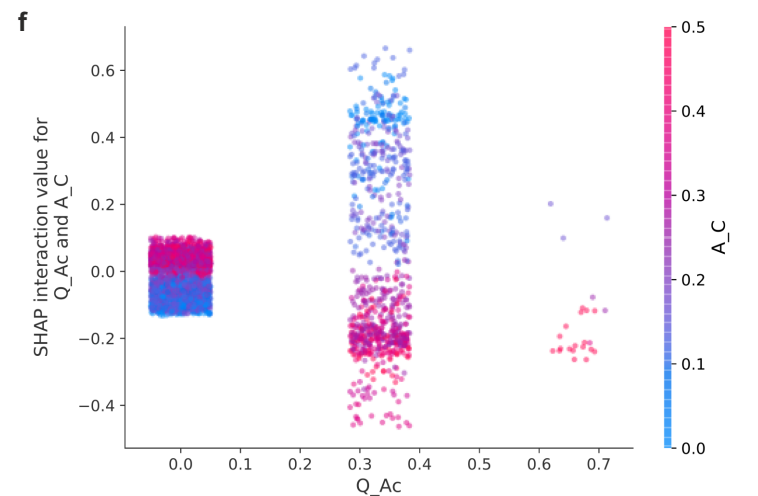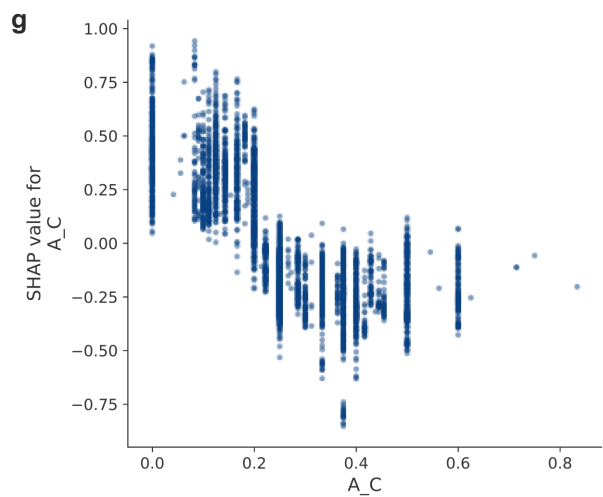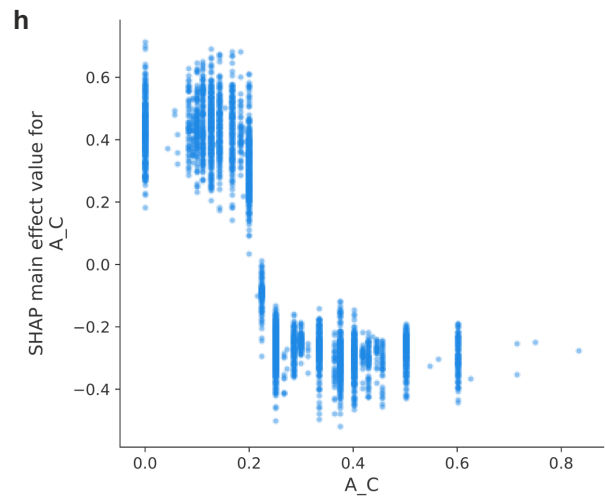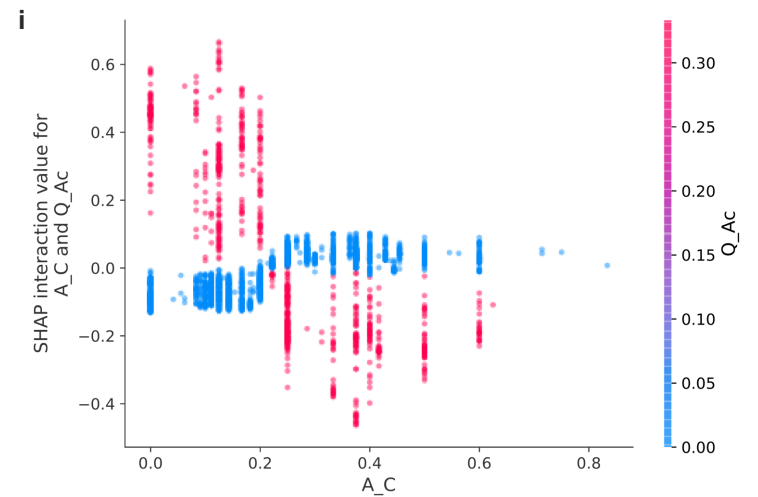

Supplement: Supplementary file 7 — sb2c00328_si_007.pdf [file sb2c00328_si_007.pdf]

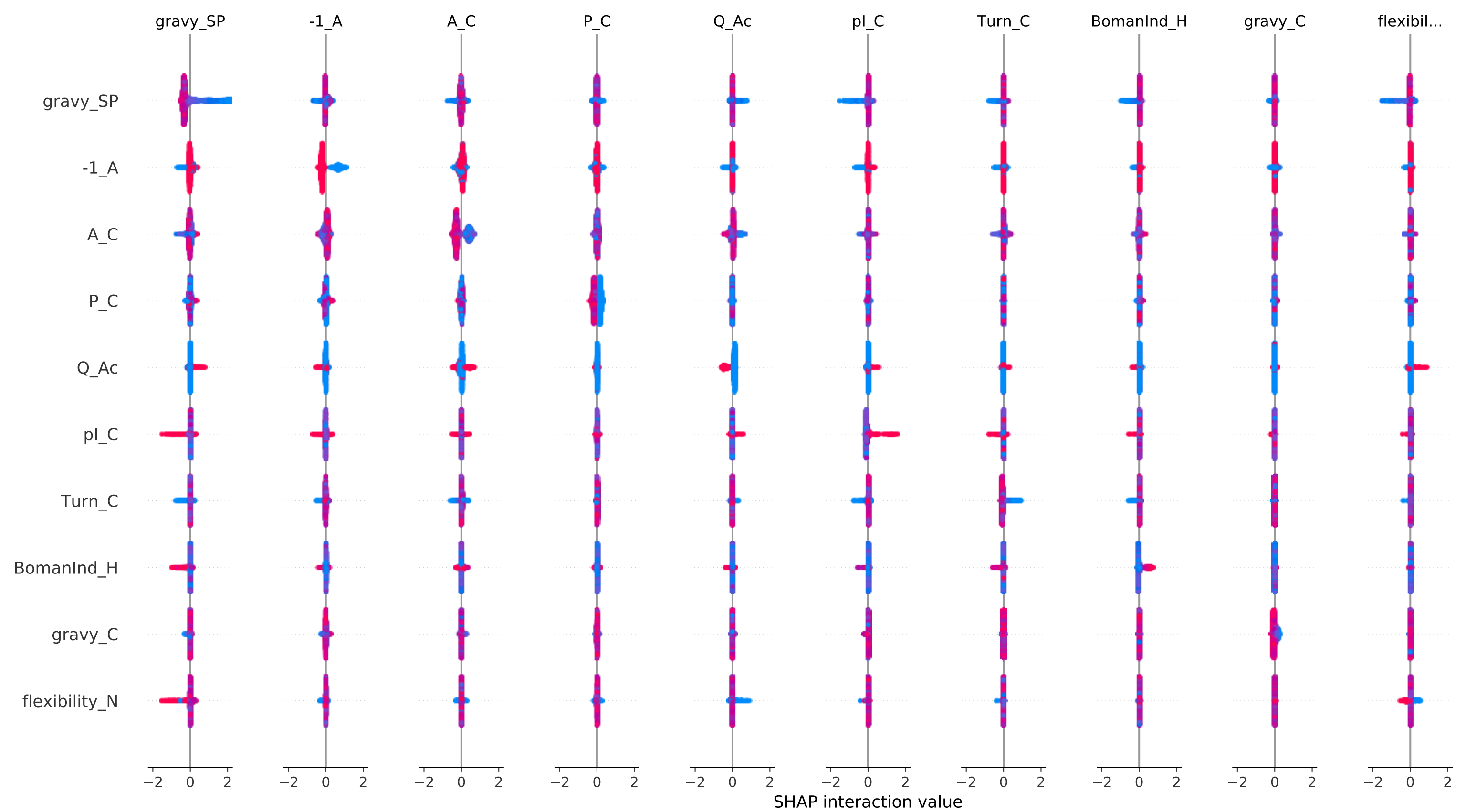

Supplement: Supplementary file 8 — sb2c00328_si_008.pdf [file sb2c00328_si_008.pdf]

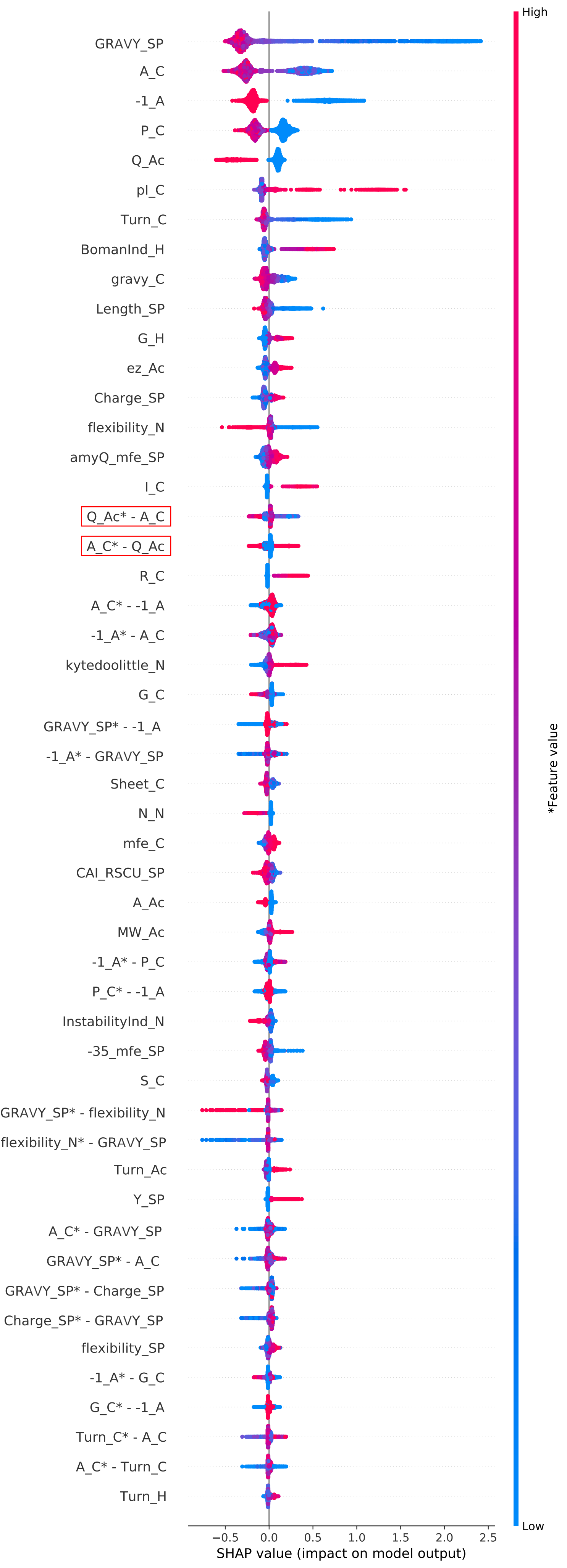

Supplement: Supplementary file 9 — sb2c00328_si_009.pdf [file sb2c00328_si_009.pdf]

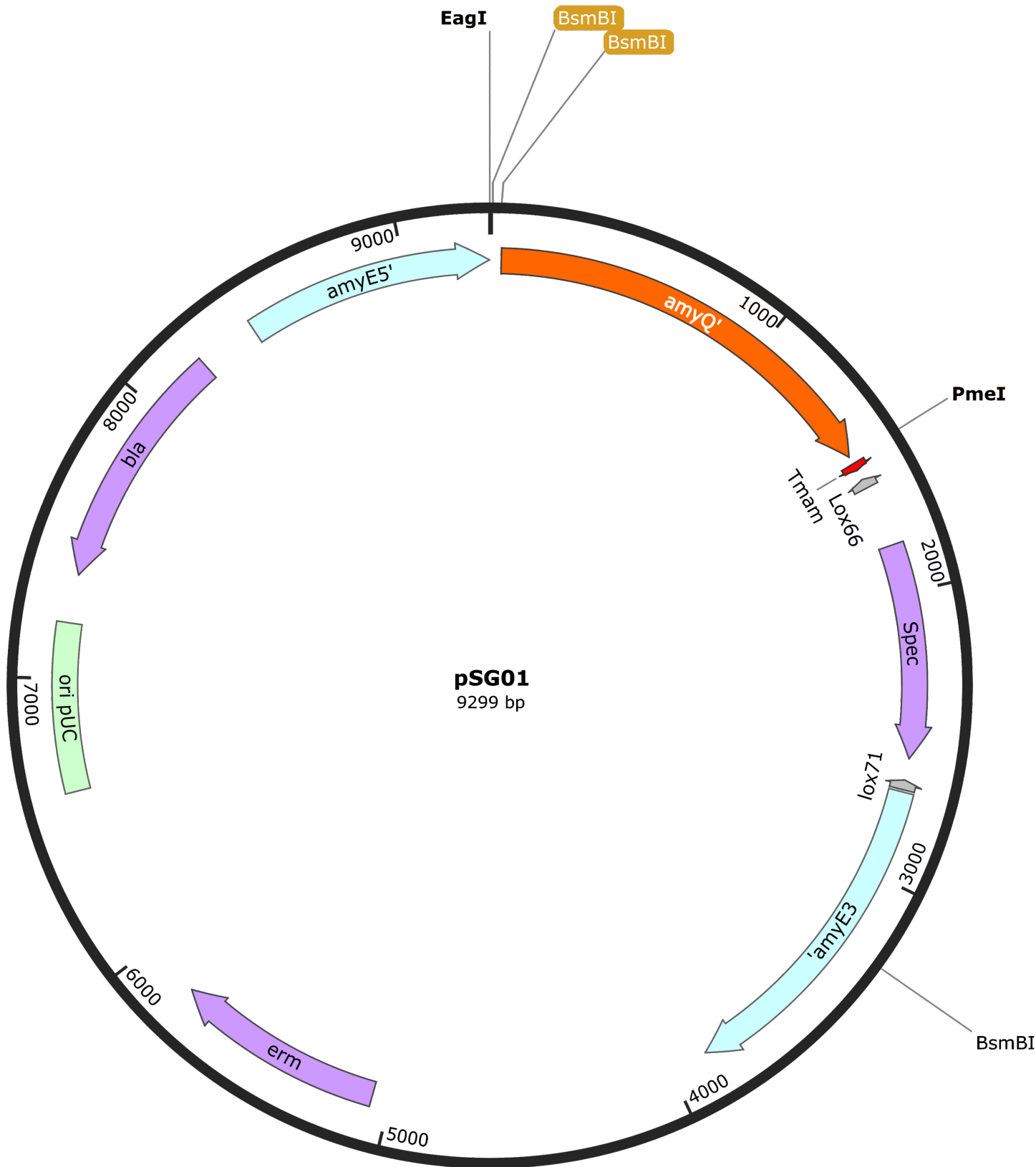

Supplement: Supplementary file 10 — sb2c00328_si_010.pdf [file sb2c00328_si_010.pdf]
